# A Comprehensive Map of Mycobacterium tuberculosis Complex Regions of Difference

**DOI:** 10.1128/mSphere.00535-21

**Published:** 2021-07-21

**Authors:** D. Bespiatykh, J. Bespyatykh, I. Mokrousov, E. Shitikov

**Affiliations:** a Federal Research and Clinical Center of Physical-Chemical Medicine, Moscow, Russia; b St. Petersburg Pasteur Institute, St. Petersburg, Russia; Washington University School of Medicine in St. Louis

**Keywords:** MTBC, *Mycobacterium tuberculosis* complex, RD, comparative genomics, deletions, large sequence polymorphisms, regions of difference, structural variants

## Abstract

Mycobacterium tuberculosis complex (MTBC) species are classic examples of genetically monomorphic microorganisms due to their low genetic variability. Whole-genome sequencing made it possible to describe both the main species within the complex and M. tuberculosis lineages and sublineages. This differentiation is based on single nucleotide polymorphisms (SNPs) and large sequence polymorphisms in the so-called regions of difference (RDs). Although a number of studies have been performed to elucidate RD localizations, their distribution among MTBC species, and their role in the bacterial life cycle, there are some inconsistencies and ambiguities in the localization of RDs in different members of the complex. To address this issue, we conducted a thorough search for all possible deletions in the WGS data collection comprising 721 samples representing the full MTBC diversity. Discovered deletions were compared with a list of all previously described RDs. As with the SNP-based analysis, we confirmed the specificities of 79 regions at the species, lineage, or sublineage level, 17 of which are described for the first time. We also present RDscan (https://github.com/dbespiatykh/RDscan), an open-source workflow, which detects deletions from short-read sequencing data and correlates the results with high-specificity RDs, curated in this study. Testing of the workflow on a collection comprising ∼7,000 samples showed a high specificity of the found RDs. This study provides novel details that can contribute to a better understanding of the species differentiation within the MTBC and can help to determine how individual clusters evolve within various MTBC species.

**IMPORTANCE** Reductive genome evolution is one of the most important and intriguing adaptation strategies of different living organisms to their environment. Mycobacterium offers several notorious examples of either naturally reduced (Mycobacterium leprae) or laboratory-reduced (Mycobacterium bovis BCG) genomes. Mycobacterium tuberculosis complex has its phylogeny unambiguously framed by large sequence polymorphisms that present unidirectional unique event changes. In the present study, we curated all known regions of difference and analyzed both Mycobacterium tuberculosis and animal-adapted MTBC species. For 79 loci, we have shown a relationship with phylogenetic units, which can serve as a marker for diagnosing or studying biological effects. Moreover, intersections were found for some loci, which may indicate the nonrandomness of these processes and the involvement of these regions in the adaptation of bacteria to external conditions.

## INTRODUCTION

The Mycobacterium tuberculosis complex (MTBC) is a group of closely related species that can cause tuberculosis (1). The members of the complex include the following Mycobacterium species: M. africanum (i.e., MTBC lineage 5 and lineage 6) (2), M. bovis (3), *M. canettii* (4), M. caprae (5), M. microti (6), *M. mungi* (7), *M. orygis* (8), M. pinnipedii (9), *M. suricattae* (10), and M. tuberculosis (i.e., MTBC lineage 1 to lineage 4, lineage 7, and lineage 8) (11–13). These microorganisms have no evidence of horizontal gene transfer between strains (14, 15), and more significantly, they are some of the examples of genetic homogeneity (99.9% nucleotide identity), except for *M. canettii* and other “smooth” mycobacteria (16). Due to their low diversity, classical genotyping techniques, such as pulsed-field gel electrophoresis or multilocus sequence typing, have proven to be practically inapt for accurate MTBC genotyping. Instead, IS*6110* restriction fragment length polymorphism (IS*6110*-RFLP) analysis, spoligotyping, and mycobacterial interspersed repetitive unit-variable number of tandem repeat (MIRU-VNTR) analysis were introduced for genotyping. The methods mentioned above are excellent for identifying microbial transmission routes, disease outbreaks, and new cases of reinfection. However, due to their high discriminatory power and, in some cases, effects of homoplasy, these methods are not entirely suitable for constructing a reliable phylogeny for MTBC members. Large sequence polymorphisms (LSPs) proved to be the best solution to this problem, until whole-genome sequencing (WGS) technologies developed further, and single nucleotide polymorphisms (SNPs) also became pertinent to MTBC genotyping. Together, these markers facilitated the determination of a cogent scenario for the evolution paths of members of the MTBC (11, 17).

LSPs, being unidirectional unique-event polymorphisms, were initially identified using whole-genome microarrays and bacterial artificial chromosome arrays (18, 19). Located in the so-called regions of difference (RDs), LSPs were attributed to deletions relative to the reference M. tuberculosis H37Rv strain, while RvDs are H37Rv-related deletions. These deletions span from several hundred base pairs to more than 10 kbp, with the largest one being 26.3 kbp long (RD^Rio^) (20). In general, RDs can be divided into phylogenetically informative and noninformative regions. The latter include PE-PPE genes, prophage regions, and regions flanked by insertion sequences. These regions are often strain specific due to variability and homologous recombination. In contrast, phylogenetically informative deletions are conservative and inherited by all descendants of the strain. Moreover, these deletions are sometimes associated with the virulence or resistance of mycobacteria (17, 21, 22).

Today, next-generation sequencing technologies have made a breakthrough in mycobacterial research. Whole-genome sequencing is routinely used to investigate tuberculosis resistance, transmission dynamics, and the population structure of MTBC organisms (23, 24). Numerous bioinformatics pipelines have been developed for this purpose, making it possible to correlate genomic data and laboratory tests. For the phylogenetic study of the MTBC, various SNP-based tools have been developed, while only a few tools have been developed for the analysis of LSPs in mycobacteria. The most prominent of these tools is RD-Analyzer, which can predict species and lineages of MTBC isolates from sequenced reads based on the presence of a set comprising 31 previously defined markers (RDs). Additionally, the authors identified 6 potential RD markers for the differentiation of M. tuberculosis lineage 4 isolates (25).

Here, we used publicly available WGS data comprising 721 MTBC strains to search for all possible deletions in the genome. Subsequently, the found deletions were correlated with a list of 187 RDs selected from 24 studies. This allowed us to describe the specificities of 79 LSPs at the species, lineage, and sublineage levels; also, some problems that may arise when analyzing them were pointed out. In addition, we provide an RDscan workflow that was designed to find deletions and predict RDs using paired-end short-read sequencing data. Validation assessment of the workflow on a collection of ∼7,000 WGS samples showed the high specificities of the identified RDs for different phylogenetic groups.

## RESULTS AND DISCUSSION

### Sample collection and phylogeny.

Genomic analysis was performed on 9,471 SRA paired-end read sets and 367 complete Mycobacterium tuberculosis complex genomes. MTBC species differentiation and phylogenetic lineage confirmation were done using SNP-based SNP-IT software (26), main lineages within M. tuberculosis were identified based on the Coll et al. (11) classification, while the Shitikov et al. (27) and Palittapongarnpim et al. (28) classifications were used to determine more specific sublineages within lineage 2 and lineage 1, respectively. After the initial quality screening, 7,094 samples belonging to all known species and lineages met the selection criteria and were used for further analysis (see Table S1 in the supplemental material). In the final data set, M. tuberculosis genomes comprised most of the samples in the collection (*n *= 6,993) and consisted mainly of lineage 2 (*n *= 2,365) and lineage 4 (*n *= 3,152) genomes, as these are the two most globally distributed M. tuberculosis lineages. It should be noted that all known lineage 2 and lineage 4 sublineages were identified among the samples used in this study. Lineage 1 members were allocated to different sublineages, with support from the work of Coll et al. (11) and the more in-depth SNP schemes of Palittapongarnpim et al. (28). According to the Coll et al. typing scheme, the analysis was unable to successfully differentiate lineage 3 sequences into well-supported sublineages (only 296 of 993 samples were differentiated at the sublineage level). Other members of the complex accounted for 127 samples. Most of these members were *M. orygis* (*n *= 32) and *M. caprae* (*n *= 22) isolates, while both *M. mungi* and *M. suricattae* had only one isolate per species.

10.1128/mSphere.00535-21.2 TABLE S1 Whole-genome sequencing data set. Download Table S1, XLSX file, 0.01 MB. Copyright © 2021 Bespiatykh et al. 2021 Bespiatykh et al. https://creativecommons.org/licenses/by/4.0/ This content is distributed under the terms of the Creative Commons Attribution 4.0 International license.

**FIG 1:**
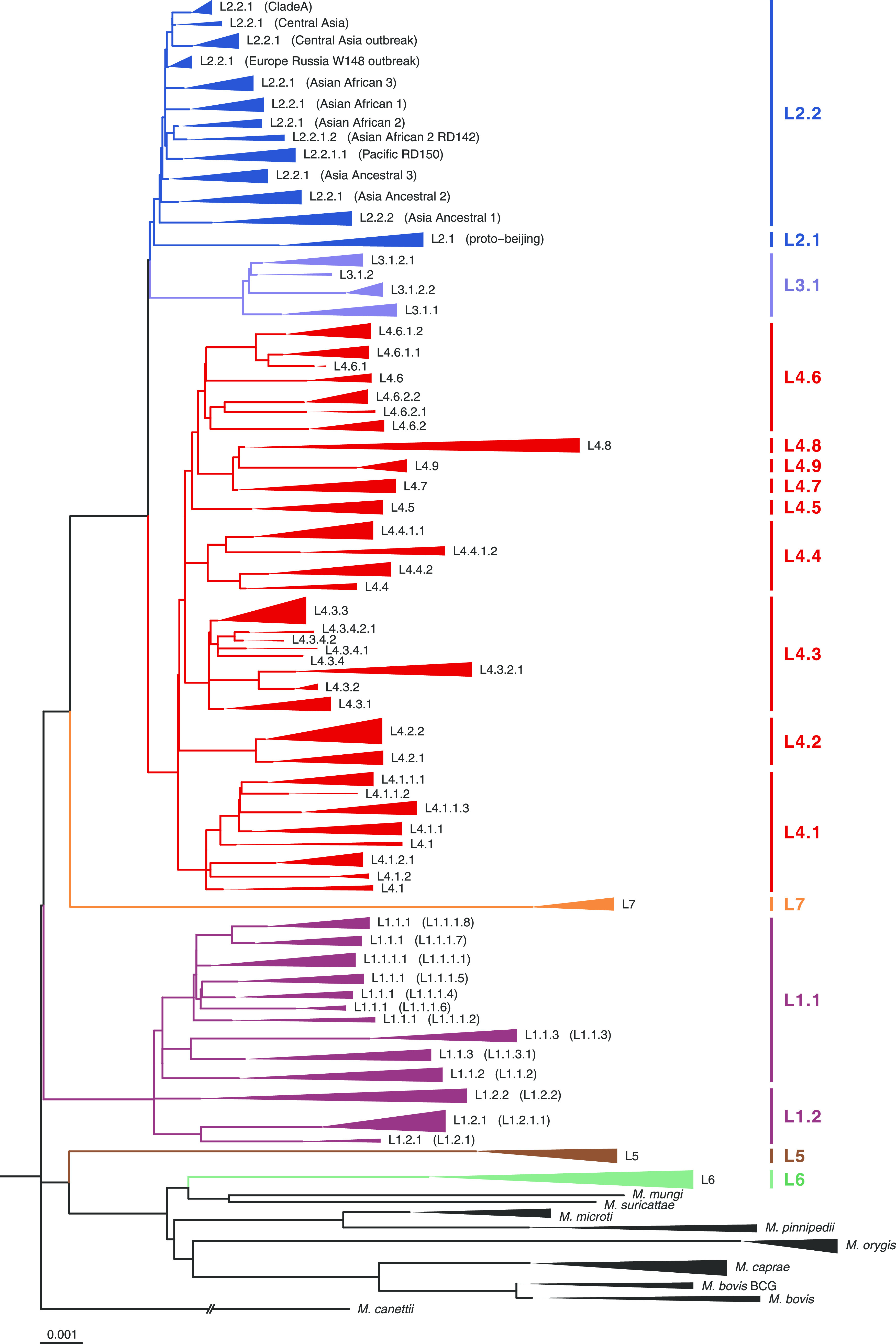
Maximum-likelihood phylogeny of MTBC species. Maximum-likelihood phylogenetic tree of 721 genomes, inferred using 30,166 nonrecombinant core genome SNPs. The scale bar indicates the number of nucleotide substitutions per site. The tree is rooted on *M. canettii* (branch length is omitted).

To equilibrate the number of samples within the groups, for further phylogenetic analysis, the data set was subsampled to contain ∼10 samples per species/sublineage (Table S2). Samples were chosen so that the final data set would include the maximum variety of samples belonging to different WGS projects. The maximum-likelihood phylogenetic tree of 721 MTBC genomes was inferred using 30,166 SNPs and rooted on *M. canettii*, the phylogenetically closest relative of the MTBC (Fig. 1). The present phylogenetic analysis demonstrated that the clustering of MTBC isolates is fairly consistent with those of previously published MTBC phylogenies (11, 23), with the advantage of this assay being that it was able to combine in one tree members of different species and keep consistent sublineage differentiations.

10.1128/mSphere.00535-21.3 TABLE S2 Samples for phylogenetic tree construction and deletion analysis. Download Table S2, XLSX file, 0.03 MB. Copyright © 2021 Bespiatykh et al. 2021 Bespiatykh et al. https://creativecommons.org/licenses/by/4.0/ This content is distributed under the terms of the Creative Commons Attribution 4.0 International license.

A phylogenetic tree showed that two main evolutionary branches can be distinguished. One clade consists of M. tuberculosis members, among which ancient and modern lineages can be discerned. The second clade contains human-adapted lineage 5 and lineage 6 members and animal-adapted MTBC species. In agreement with the previously published study, animal-adapted species can be divided into four clades: A1 (*M. suricattae*, *M. mungi*, chimpanzee bacillus, and “Dassie” bacillus [chimpanzee and “Dassie” bacilli are not included in this study]), A2 (*M. microti* and *M. pinnipedii*), A3 (*M. orygis*), and A4 (*M. caprae* and M. bovis) (23).

### Deletion discovery in MTBC genomes.

**FIG 2:**
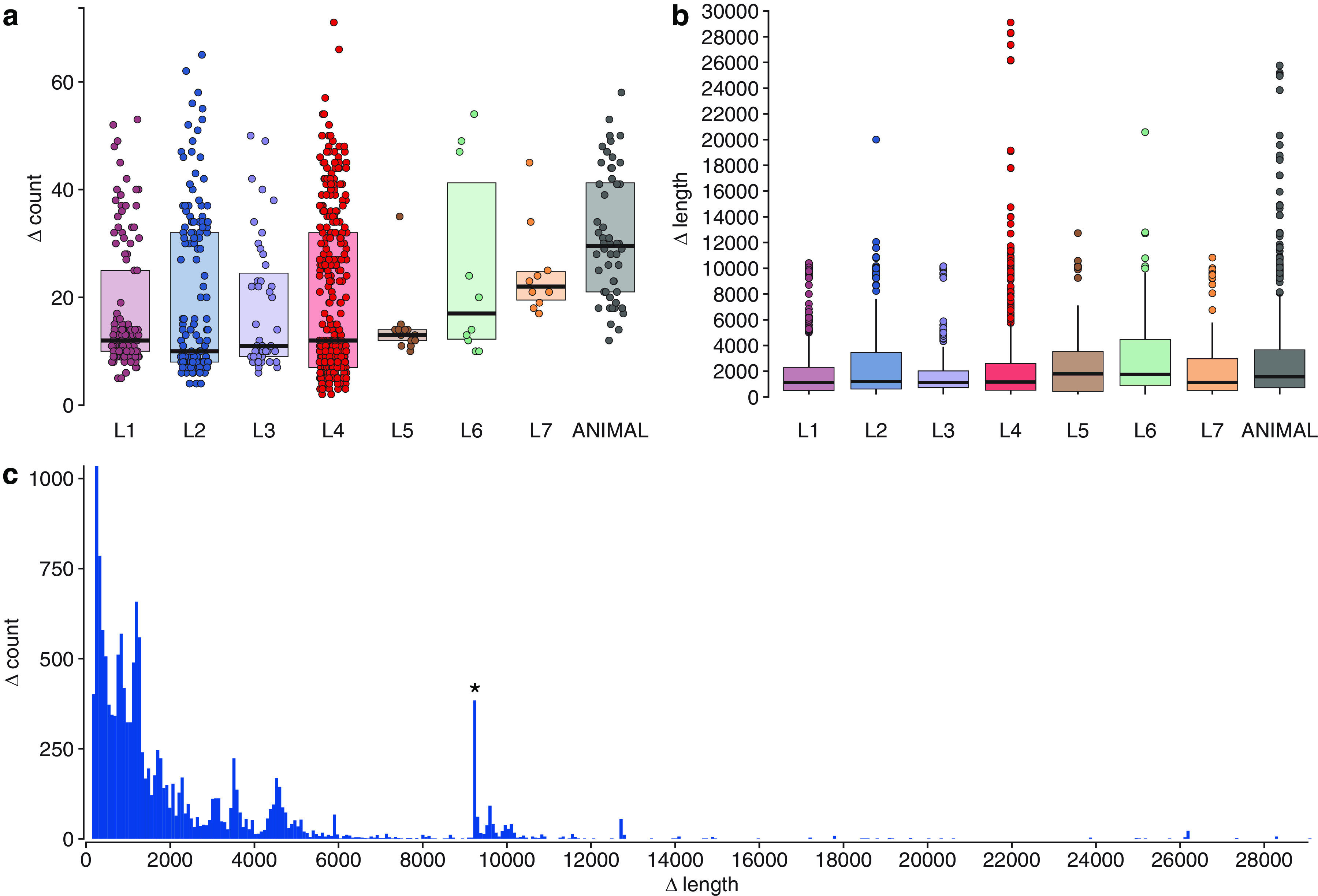
Characteristics of deletions in MTBC samples. (a) Deletions per genome distribution among MTBC strains. Each point represents an individual sample. The *y* axis indicates the number of deletions per sample. The box represents the interquartile range that contains 50% of the values. A line across the box indicates the median. (b) Deletion length distribution among lineages. The *y* axis shows deletion length, and points represent outliers. The boxes indicate upper and lower quartiles, and the horizontal lines mark the medians. Whiskers indicate maximum and minimum values, excluding outliers. (c) Size distribution of deletions among all samples. The outlier peak is marked with an asterisk. L1 to L7, lineages 1 to 7.

For the detection of deletions, the same set of paired-end short-read samples belonging to all main MTBC lineages and sublineages was used (*n *= 721). In total, 14,471 deletions were found in the data set, the largest of which was 29,106 bp (this is an RD^Rio^ deletion, which has been falsely said to be 2,800 bp longer due to poor coverage in that region of a specific sample); on average, 20 (SD = 14) deletions per genome were discovered (Fig. 2a and b). The outlier peak was discovered among deletion lengths at 9,238 bp (Fig. 2c), which corresponded to deletions in the CRISPR locus.

The largest average length of deletion per sample was found in lineage 6 isolates (3,209 bp per sample), followed by animal-adapted species (3,110 bp per sample), and the highest frequency of deletions per genome was observed in animal-adapted genomes (31 deletions per sample, SD = 12) (Fig. 2a).

Fisher’s exact test for enrichment estimation of 969 samples affected by deletions in genes, based on the TubercuList database annotation (http://genolist.pasteur.fr/TubercuList/), identified two overrepresented functional categories comprising 165 genes (*P < *0.05): “PE/PPE families” (*P = *1.89E–39; *n *= 118) and “Insertion sequences and phages” (*P = *0.01; *n *= 47). The second enrichment test was performed with annotation, based on a gene’s essentiality for the bacterial life cycle (29); this test showed that the only enriched category in the investigated gene set is “nonessential” (*P = *1.69E–24; *n *= 838). However, two essential genes disrupted by deletions were present in the call set. The Rv1122 gene (*gnd2*; 6-phosphogluconate dehydrogenase) was partially deleted in some members of lineage 4.6.2 (5 samples), while the Rv2017 gene (transcriptional regulator) was disrupted in a number of independent deletion events.

### Species-, lineage-, and sublineage-specific RDs.

A comprehensive list of RDs, based on previously published studies, was made to correspond to deletions from this study with already-well-described loci (Table S3). Additionally, M. tuberculosis H37Rv-related RvD1-5, TbD1, and RD900 deletions were used in the current study (17, 30, 31). A total of 187 RDs were included; out of these, 61 belonged to animal-adapted regions, and the remaining regions corresponded to M. tuberculosis.

10.1128/mSphere.00535-21.4 TABLE S3 Complete list of the regions of difference. Download Table S3, XLSX file, 0.02 MB. Copyright © 2021 Bespiatykh et al. 2021 Bespiatykh et al. https://creativecommons.org/licenses/by/4.0/ This content is distributed under the terms of the Creative Commons Attribution 4.0 International license.

The analysis revealed two types of deletions that agree with the results of previously published studies (17). The first type was affiliated with repeat sequences or mobile genetic elements, such as prophages and insertion sequences. The name of these deletions is collective, and their breakpoints may vary both at the species level and with strains of the same lineage level. The latter significantly reduces the putative differential capabilities of particular RDs. Some of the best-known types of such regions are RD3 (also known as DS5 and RD149) and RD11 (also known as DS10 and RD198a), related to prophage sequences phiRv1 and phiRv2, respectively. These elements are deleted among different species and lineages of the MTBC, which points to the independence of these deletions and the instability of the affected genomic regions. Other examples of these deletions are RD6, containing IS*1532*, or RD5, RvD2, RvD3, RvD4, RvD5, and RD152, related to deletions in IS*6110* flanking regions or recombination events between repeats. MID3 and MID4 also belong to this type of deletion and are associated with repetitive sequences in MTBC genomes.

The most notable RD related to this type of deletion is RD5. This deletion is often mentioned in the literature, and it has been reported to contribute to the virulence of the MTBC members (32). Initially, RD5 was described when M. tuberculosis H37Rv was compared with M. bovis BCG (31) and is often found (with various positions) among different M. tuberculosis strains. However, in the case of animal-adapted strains, specific deletion breakpoints are indicated (Fig. S1). M. bovis and *M. caprae* have the same breakpoints of deleted RDs, which can indicate the presence of this deletion in ancestor species, whereas, in the case of *M. orygis*, a wide variety of deletions was discovered with slightly different 5′-end positions, while the 3′ end corresponded to previously described RD5^oryx^ (33). For *M. microti*, the presence of RD^mic^ could not be confirmed due to the fact that the analyzed genomes, as well as the reference genome (GenBank accession no. CP010333.1), were intact at this locus. Only MTBC strains that cause tuberculosis in voles have been described to have this deletion, whereas human strains do not (6). *M. pinnipedii* as well as *M. microti*, being a member of animal-adapted clade A2, was also intact at the RD5 locus. It is not yet possible to judge the diversity of RD5 in *M. mungi*, *M. surricattae*, and “Dassie” bacillus due to the small number of publicly available strains. Nonetheless, it can be assumed that RD5^das^ (34) and RD5^sur^ (35) are identical deletions.

10.1128/mSphere.00535-21.1 FIG S1 Comparison of the RD5 regions. RD5 region deletions among different MTBC species are presented relative to M. tuberculosis H37Rv. Download FIG S1, EPS file, 0.5 MB. Copyright © 2021 Bespiatykh et al. 2021 Bespiatykh et al. https://creativecommons.org/licenses/by/4.0/ This content is distributed under the terms of the Creative Commons Attribution 4.0 International license.

**FIG 3:**
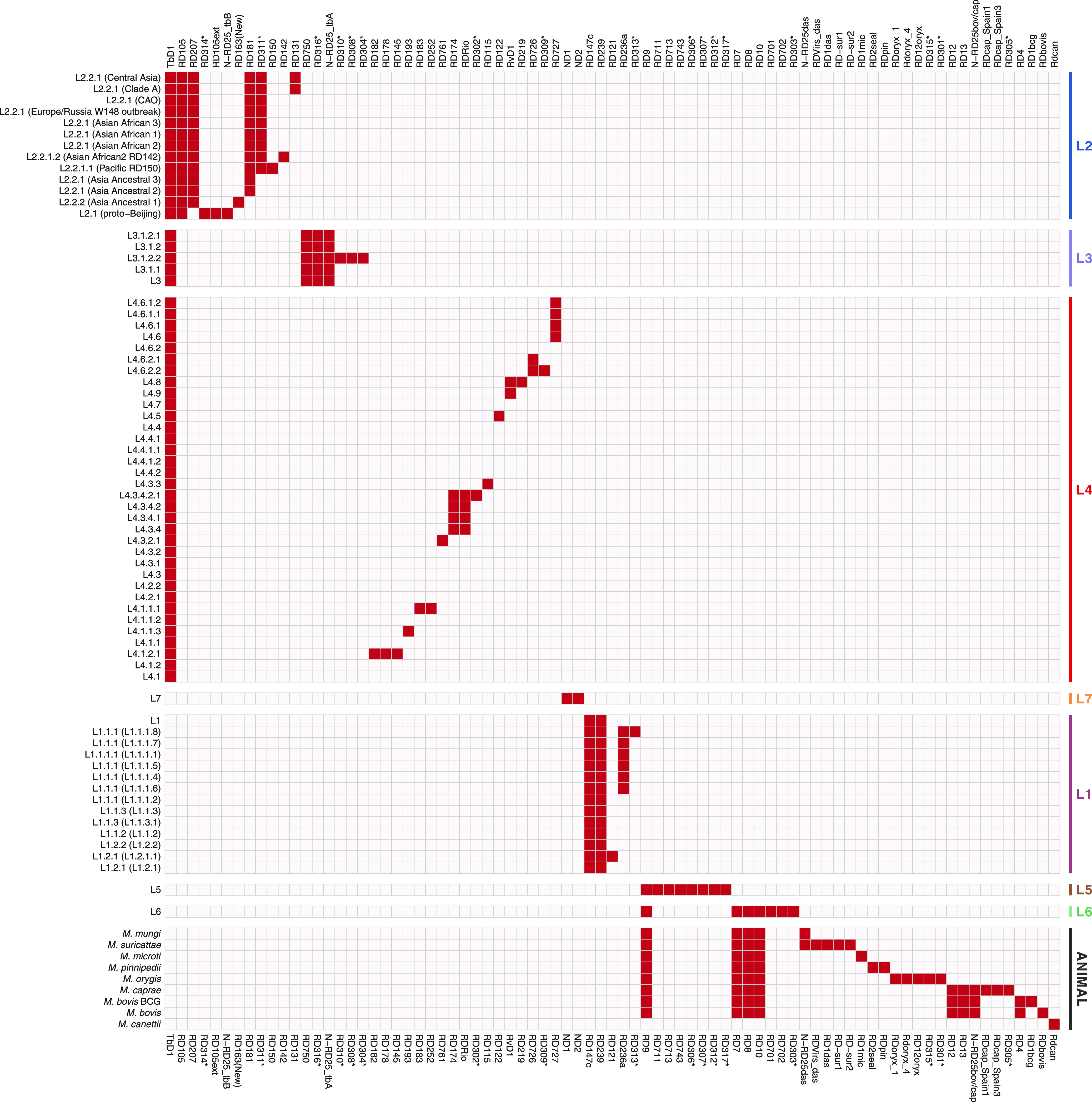
RD distribution across main MTBC phylogenetic units. RDs present in M. tuberculosis H37Rv and absent in the studied lineages are in red (RvD1 and TbD1 are exceptions). The rows represent lineages within M. tuberculosis or MTBC species, and each column is a specific region of difference. Lineages and species in rows are arranged according to their phylogenetic relationship based on SNP analysis. RDs found in this study are marked with asterisks.

The second type of deletion corresponds to RDs, the flanking regions of which do not contain repeat sequences. As a result, we found 79 such deletions, which were characteristic of phylogenetic units derived from SNP analysis. It should be noted that characteristic deletions were found for all lineages, as well as for most sublineages of the complex. Out of 79 RDs, 33 were specific to M. tuberculosis lineages 1 to 4 and lineage 7 and correlated with well-known RDs; also, 10 new deletions that had not been described prior to this study were found (Fig. 3). Seven out of the 10 new deletions were sublineage specific, while the other 3 (RD311, RD316, and RD306) could be found across different sublineages within the lineage. The small deletion RD311 (213 bp), which leads to Rv2434c inactivation, was found among all modern Beijing strains and can serve as an additional marker for the detection of strains belonging to this group (exceptions are bmyc26 group strains, belonging to the ancient Beijing genotype family, as they do not bear a deletion in this locus [data not shown]). The RD316 (1,297 bp) deletion, resulting in the loss of the Rv3516 and Rv3517 genes, is specific to all members of lineage 3. Rv1179c and was truncated by RD306 (256 bp), which was specific to lineage 4.4.1.1 and lineage 4.4.1.2 sublineages.

For the second phylogenetic clade, comprising M. tuberculosis lineage 5, lineage 6, and animal-adapted MTBC species, 29 previously described and 7 novel RDs were found (Fig. 3). Newly described RD307, RD312, and RD317 were found in lineage 5 samples. RD303 (375 bp), affecting the Rv0267 (*narU*) gene, was specific to lineage 6. RD301 and RD315 were unique to *M. orygis*, while the RD305 deletion was specific to *M. caprae*.

### Distribution of specific deletions across the H37Rv genome.

The largest number of RDs specific for phylogenetic units (*n *= 79) was located in independent regions of the genome, while a slight symmetry in the distribution of deletions relative to the origin of replication was observed. It should also be noted that a slightly higher number of specific deletions than in other regions was found in the 1.3- to 3.0-Mbp genomic region, which is consistent with previously published findings (36).

**FIG 4:**
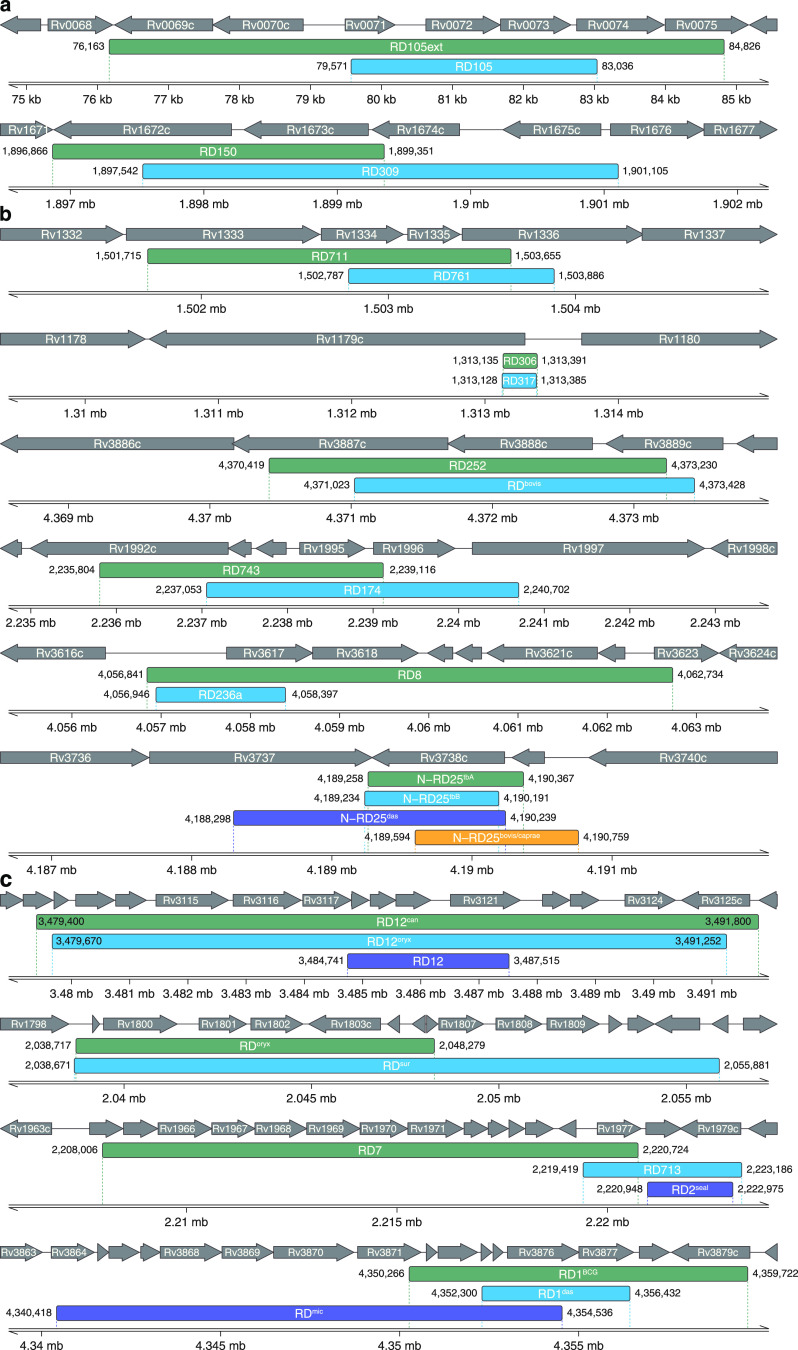
Overlapping RDs in different MTBC members. Deletions relative to the M. tuberculosis H37Rv genome are shown in green, blue, purple, and orange; gray arrows indicate genes. Overlapping RDs within Mycobacterium tuberculosis lineages (a), between M. tuberculosis (lineages 1 to 4 and lineage 7) and other MTBC members (b), and within M. tuberculosis lineage 5 and lineage 6 and the animal-adapted clade (c).

Overlaps spanning full or partial deletion lengths were found across 29 deletions when one of the deletions intersected another or was located directly inside the largest one (Fig. 4). For M. tuberculosis, one such pair of overlapping deletions is RD105 and RD105ext. RD105ext is specific to the members of proto-Beijing lineage 2, while RD105 is a classic marker for all other lineage 2 members. An RD150 deletion affecting four genes (Rv1671, Rv1672c, Rv1673c, Rv1674c) was found among lineage 2.2.1.1 (Pacific RD150) isolates, and it overlaps the larger RD309 deletion, specific for lineage 4.6.2.2 (Fig. 4a).

For six regions, the intersection of RDs specific for two large evolutionary branches was found (Fig. 4b). Lineage 4.3.2.1-specific RD761 has breakpoints similar to those of lineage 5-specific RD711. RD306 (lineages 4.4.1.1 and 4.4.1.2) is almost exactly the same as RD317 (lineage 5). RD252 and RD^bovis^ are specific for lineage 4.1.1.1 and M. bovis, respectively, and also have similar breakpoints. Lineage 4.3.4-specific RD174 intersects with RD743, specific to lineage 5; both deletions affect the Rv1995 and Rv1996 genes, belonging to the “Growth-Advantage” gene group (29). Another overlapping pair contains RD8 (lineage 6 animal-adapted species) and RD236a, which is specific for some lineage 1 sublineages. The N-RD25 deletion was found in different Mycobacterium genus members: N-RD25^tbA^ was found to be specific for many lineage 3 strains, N-RD25^tbB^ for lineage 2.1 (proto-Beijing), N-RD25^bovis/caprae^ for M. bovis*/M. caprae*, and N-RD25^das^ for *M. mungi*, *M. suricattae*, and “Dassie” bacillus.

Ten overlapping RDs were identified in phylogenetic clades, including M. tuberculosis lineage 5, lineage 6, and animal-adapted species (Fig. 4c). One such deletion is RD12; it was originally identified as specific for M. bovis (31), but the deletion was also found in *M. caprae*. RD12 is overlapped by a larger RD12^oryx^ deletion specific for *M. orygis*, as well as comparably sized RD12^can^, which was found in almost all the *M. canettii* samples included in this study. One more overlapping pair is RD^sur1^ and RD^oryx_1^, in which RD^sur1^ is much larger and affects 15 genes versus 8 genes affected by RD^oryx_1^. Consecutively, for the RD7 region (lineage 6 plus animal-adapted species), a small intersection with RD713, specific for lineage 5, was found. It was also discovered that RD713 completely overlaps RD2^seal^, which is specific for *M. pinnipedii*. In addition, the complexity of this region lies in the fact that some BCG vaccine strains also contain a large RD2 deletion in this region (not shown in Fig. 3 and 4, since the marker is detected in only some strains [37]). The last RD related to this group was RD1, which was also originally identified in vaccine strains and is thoroughly described in previously published studies in connection with its virulence role (34).

### RDscan workflow testing.

To assess the performance of the RDscan pipeline, the following state-of-the-art structural variant (SV) detection tools were used: delly (v.0.8.7) (38), TIDDIT (v.2.12.1) (39), and breseq (v.0.35.7) (40). These tools were chosen because, in our practice, they produced the best results on haploid genomes detecting large deletions from mapped WGS reads.

For this benchmarking, we used *M. microti* strain OV254 (ENA database run accession no. ERR027294). Strain OV254 has been reported to harbor deletions in RD1^mic^, RD3, RD7, RD8, RD9, RD10, MiD3, RD11 (partial), MiD1, RD5^mic^, and MiD2 RDs (41). In addition, we manually curated the *M. microti* OV254 deletions using samplot (v.1.1.6) (https://github.com/ryanlayer/samplot) and IGV (v.2.9.4) (42). Consequently, the RD236a deletion was discovered, as well as some non-RD-specific deletions.

To compare the mentioned tools with RDscan, we used sensitivity (equation 1), precision (equation 2), and F_1_ score (equation 3) as defined in the following equations:

(1) $$\text{sensitivity}=\frac{\text{TP}}{\text{TP}+\text{FN}}$$

(2) $$\text{precision}=\frac{\text{TP}}{\text{TP}+\text{FP}}$$

(3) $${\text{F}}_{\text{1}}=2\times \frac{\text{sensitivity×precision}}{\text{sensitivity}+\text{precision}}$$

where TP means true positive, the number of correctly identified deletions, FP means false positive, the number of nondeleted regions that were incorrectly identified, and FN means false negative, the number of deletions that were incorrectly rejected. The harmonic mean between precision and sensitivity (F_1_ score) was used to determine the tool with the best balance between sensitivity and precision.

RDscan showed the highest sensitivity and F_1_ score among all tools (Table 1). TIDDIT had the best precision (90%) and second-best performance (52%) but lacked in sensitivity compared to RDscan and Delly. In the case of known RDs in the genome, RDscan did not register partial deletions in RD11, Delly did not find deletions in MiD1, RD5^mic^, and MiD2, breseq did not find deletions in RD3, MiD3, MiD1, RD5^mic^, and MiD2, and TIDDIT did not find deletions in RD3, RD11, MiD1, RD5^mic^, and MiD2.

**TABLE 1:** Comparisons of different tools for the *M. microti* OV254 genome^*a*^

Tool	Sensitivity	Precision	F_1_
RDscan	**0.920**	0.442	**0.597**
TIDDIT	0.375	**0.900**	**0.529**
Delly	0.407	0.180	0.250
breseq	0.280	0.467	0.350

a Values in bold are the best results for the corresponding evaluation criteria.

This performance benchmark shows that RDscan surpasses other methods in terms of overall performance (59%) and sensitivity (92%).

A more extensive analysis was performed to validate the efficacy of RDscan in inferring large deletions. The pipeline was run on an initial data set comprising 7,094 paired-end samples (Table S1). Putative regions of difference discovered with RDscan were compared with a database containing currently well-described RDs. As a result, the presence of all RDs discovered in a smaller data set was confirmed. In addition, for some regions, we noted some specificities that must be taken into account in further analysis.

**FIG 5:**
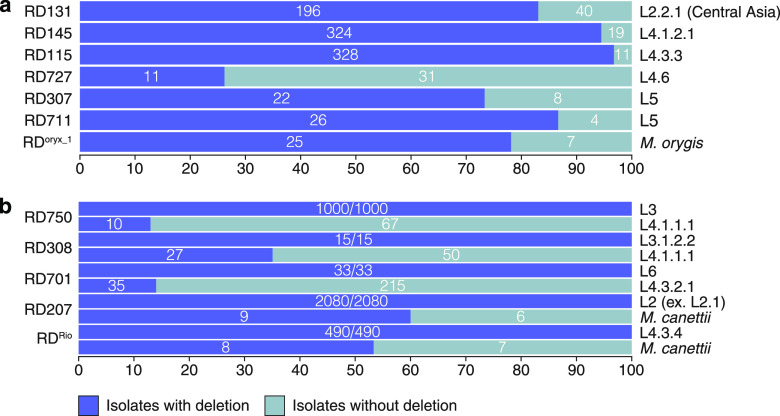
Ambiguous and interlineage RDs. Stacked bar plots showing the percentages of studied isolates with (blue) and without (gray) particular deletions. (a) Ambiguous RDs; (b) interlineage RDs.

First, for 7 out of 79 RDs, it was found that not all members of the group contained the analyzed deletion (Fig. 5a). This fact suggests that the SNP markers underlying modern typing are, in this case, phylogenetically earlier than the analyzed deletions. For example, RD115, RD145, RD131, and RD727 were not found in some samples of lineage 4.3.3, lineage 4.1.2.1, lineage 2.2.1 (Central Asia) and lineage 4.6, respectively. For RD711, specific for lineage 5, the ratio of intact strains with respect to this locus was 13.4%, which is consistent with previously published observations (43). Moreover, the RD307 deletion was found even within isolates with RD711 deleted, which was not previously described. The final notable RD is RD^oryx_1^, the deletion of which was found in 25/32 samples of *M. orygis*. In this case, the absence of the deletion should be attributed to the incorrectly rejected false-negative results of the pipeline, which were detected during manual data curation. It should be noted that part of this region is still present in these *M. orygis* samples but in a different region of the genome, which leads to false results.

Another notable group contained RDs whose specificities were reduced due to the detection of similar deletions in other populations of mycobacteria (>10% of the sublineage/lineage/species). In total, five such loci were identified (Fig. 5b). It should be noted that the boundaries of deletions for specific RDs never coincided with nonspecific LSPs, which once again emphasizes the independence of recombination events that have already occurred. The most prominent of these regions were RD701, RD750, and RD207, specific to lineage 6, lineage 3, and lineage 2 (except lineage 2.1), respectively. RD701 was deleted in 11% of lineage 4.3.2.1 samples, RD750 was deleted among lineage 4.1.1.1 strains, while RD207 was deleted in a significant number of *M. canettii* strains. RD^Rio^, specific to lineage 4.3.4, is seldom detected in other mycobacteria; similar deletions were detected only among some *M. canettii* strains. However, this region is generally unstable and contains many repeats and corresponding deletions. For example, MID3 overlaps significantly with RD^Rio^ and is found in lineage 4.6.2.2, *M. pinnipedii*, *M. microti*, and *M. canettii*.

The third and most significant group consisted of overlapping RDs, which were described earlier. According to the RDscan results, only the following RD combinations can be used for reliable strain differentiation: RD105/RD105ext, RD761/RD711, RD236a/RD8, and RD1^BCG^/RD1^das^/RD1^mic^.

### Conclusions.

Prior in-depth studies of RDs have provided a fundamental understanding of the evolutionary history of MTBC members. Furthermore, the great majority of studies may be divided into two types. The first type focuses on the investigation of phylogenetic interactions of species within the complex, where only the most significant regions are taken into account for M. tuberculosis. The second type concentrates on the search for deletions within M. tuberculosis without regard to other members of the complex.

Here, we closed the gap by collating all the previously described RDs and analyzing them on a sample set representing the complete variety of MTBC members. Although we expectedly confirmed the convergence of the main classifications at the SNP and RD levels, we also described the RDs that may overlap; in addition, we showed that some RDs are not always specific to the sublineages. It is important to note that our method has its drawbacks. Due to the high number of M. tuberculosis strains in the NCBI database, few other MTBC members were used in this study; this means that the found deletions, especially those that are sublineage specific, should be treated with caution. Another issue is that we were unable to classify lineage 3 samples, and most of the deletions found were characteristic of the entire lineage. The third disadvantage of this study is that all new deletions found have been identified *in silico* and require further experimental verification.

To alleviate the aforementioned disadvantages and facilitate the work with comprehensive genomics data, we have created a pipeline that can search for deletions in MTBC genomes. Its main advantage is that it is able to correlate deletions with a list that can be modified to meet the needs of a particular task. The herein-proposed RDscan pipeline can be used as a practical tool to rapidly infer known deletions in RDs and thus differentiate the strains, as well as describe the new deletions associated with the evolution of the pathogen.

## MATERIALS AND METHODS

### Data set.

For the analysis, a collection of 9,471 draft MTBC genomes publicly available in the NCBI SRA archive (https://www.ncbi.nlm.nih.gov/sra) was used. Target SRA files were downloaded with the prefetch (v.2.10.3) tool from the SRA Toolkit (http://www.ncbi.nlm.nih.gov/books/NBK158900/). Parallel-fastq-dump (v.0.6.6) (https://github.com/rvalieris/parallel-fastq-dump) was used to extract paired-end FASTQ reads from SRA files. A quality control (QC) check on all acquired reads was done with FastQC (v.0.11.9) (http://www.bioinformatics.babraham.ac.uk/projects/fastqc/). In addition, 367 complete MTBC genomes were obtained from the NCBI Nucleotide database (https://www.ncbi.nlm.nih.gov/nucleotide/).

### SNP calling and lineage typing.

SNPs against the M. tuberculosis H37Rv (GenBank accession no. NC_000962) and *M. canettii* (NC_015848.1) genomes were inferred using the Snippy pipeline (https://github.com/tseemann/snippy). NUCmer (v.3.1) (44) was used to call SNPs from complete MTBC genomes. BCFtools (v.1.9) (https://github.com/samtools/bcftools) was used to collect statistics on called variants. Mapping quality was assessed with Qualimap (v.2.2.2) (45). MultiQC (v.1.9) (46) was used for QC report aggregation. Only samples with at least 80% of mapped reads and with a ≥50× mean coverage were used for further analysis. Lineage/sublineage typing was performed using TB-Profiler (v.2.8.12) (47), KvarQ (v.0.12.2) (48), BioHansel (v.2.4.0) (https://github.com/phac-nml/biohansel), SNP-IT (v.1.0.0) (26), and in-house python scripts used with various previously published typing schemes (11, 27, 28).

### Phylogenetic analysis.

A core SNP alignment was produced with snippy-core (v.4.6.0) (https://github.com/tseemann/snippy). Gubbins (v.2.4.1) (49) was used to filter out recombinant regions from the alignment. The resulting alignment was cleaned to include only core polymorphic sites with SNP sites (50). Cleaned core alignment was used to construct a phylogenetic tree via RAxML-NG (v.1.0.1) (51) using the GTR+G model and 100 bootstrap iterations; the tree was rooted on *M. canettii* (GenBank accession no. NC_015848.1). The tree was visualized with the ggtree (v.2.0.2) (52) package for R (v.4.0.2) (53).

### Structural variants detection.

To detect regions with structural variants (SVs), i.e., large deletions [>200 bp], regions with low coverage and with a length of ≥100 bp were extracted with covtobed (v.1.2.0) (54); further regions that were located within 1,500 bp of each other were merged with bedtools (v.2.29.2) (55). The SURVIVOR (v.1.0.7) (56) tool was used to convert the resulting .bed files with SV breakpoints to variant call format (VCF) and to further merge these files into a single multisample .vcf file. The resulting .vcf file was annotated with SnpEff (v.4.1l) (57). Identified RDs were detected by calculating median coverage in these regions with mosdepth (v.0.3.1) (58) and dividing it by median coverage of the full mapping length. A 5% threshold was used to determine whether RD regions are present in the sample. GNU parallel (v.20161222) (59) was used to speed up some parts of the analysis. Small deletions of <200 bp and deletions larger than 30,000 bp were eliminated from the analysis to reduce the number of false-positive calls. All calls were manually curated using Integrative Genomics Viewer (IGV) (v.2.8.4) (42) and samplot (v.1.0.20) (https://github.com/ryanlayer/samplot). Breakpoints were curated using *de novo*-assembled MTBC genomes; for the *de novo* assembly, genomes were cleaned of low-quality reads and adapters with fastp (v.0.20.1) (60) and assembled using Unicycler (v.0.4.8) (61). Only high-confidence deletions were kept for downstream analysis. Plots were generated within R (v.4.0.2) (53) using the ggplot2 (v.3.3.2) (62), cowplot (v.1.1.0) (https://CRAN.R-project.org/package=cowplot), Gviz (v.1.32.0) (63), lemon (v.0.4.5) (https://CRAN.R-project.org/package=lemon), and ComplexHeatmap (v.2.7.6.1004) (64) packages.

### RDscan workflow.

An RDscan workflow was designed for deletion discovery in MTBC species using paired-end short-read FASTQ files. RDscan is implemented as a custom Snakemake (65) workflow. The workflow can be divided into two blocks; the first block finds all putative deletions, while the second scans whether already-known RDs are present in the sample. Concisely, reads are mapped to the M. tuberculosis H37Rv (GenBank accession no. NC_000962) reference genome using BWA-MEM (66). After the mapping step is finished, .bam files are indexed with SAMtools (67). Next BEDTools and SAMtools are used to generate .bed files with per-sample breakpoints by searching for regions with low coverage. Then SURVIVOR, GATK (68), and BCFtools are used to convert .bed files with putative deletions to .vcf files and prepare them for further steps. Duphold (69) is then used to calculate fold change for the deletion depth relative to flanking regions; the resulting .vcf files are filtered with BCFtools by minimum and maximum lengths (200 bp < deletion < 30,000 bp) and a duphold flank fold change (DHFFC) of <0.1. .vcf files from multiple files are then merged into a single call set using SURVIVOR and annotated with SnpEff; the resulting cohort .vcf file containing all deletion calls is transformed into a table using GATK, and putative RD regions are annotated. The second block starts with coverage computation using mosdepth in 79 specific RD regions identified and curated in this study. Lastly, the ratio of read depth in RD regions to full reference length depth is calculated, and results are merged into a single data frame; human-readable tables are then generated.

### Functional enrichment analysis.

To determine the significance of genes affected by deletions, functional categories from the TubercuList database (http://genolist.pasteur.fr/TubercuList/) and a custom database based on M. tuberculosis gene essentiality (29) were used. Enrichment scores of functional categories were obtained using Fisher’s exact test in R (v.4.0.2).

### Data availability.

RDscan is an open-source software available in the GitHub repository at https://github.com/dbespiatykh/RDscan.
